# Supplementary material for: Overexpression of Nitrate Transporter OsNRT2.1 Enhances Nitrate-Dependent Root Elongation
Source: Genes (Basel). 2019 Apr 9;10(4):290. doi: 10.3390/genes10040290 (PMC6523718; doi:10.3390/genes10040290)
Supplement: Supplementary file 1 [file genes-10-00290-s001.zip › Supplementary Materials.docx]

**Table S1.** Primers used for qRT-PCR.

**Table S2.** The average data statistics of the ^15^NO_3_^–^ influx rate in roots of WT and *OsNRT2.1* transgenic lines under 0.5-mM ^15^NO_3_^–^ conditions with NPA and without NPA. Significant differences are indicated by different letters (*p* < 0.05, two-way ANOVA).

**Figure S1.** Rice seed size. (**A**) Grain length, (**B**) grain width, (**C**) 1000-grain weight, (**D**) total nitrogen concentration. Error bars: SE (*n* = 10). Significant differences between transgenic lines and WT are indicated by different letters (*p* < 0.05, one-way ANOVA).

**Figure S2.** Plant growth of WT and *OsNRT2.1* transgenic lines under 0.5-mM NH_4_^+^ or NO_3_^–^ conditions. Rice seedlings were grown in a quarter concentration of nutrient solution containing 0.5 mM of NH_4_^+^ or NO_3_^–^ conditions at the beginning. The phenotype of the transgenic lines under (**A**) 0.5-mM NH_4_^+^, or (**D**) 0.5-mM NO_3_^–^ conditions bar = 5 cm; length of shoot under (**B**) 0.5-mM NH_4_^+^ or (**E**) 0.5-mM NO_3_^–^ conditions; length of root under (**C**) 0.5-mM NH_4_^+^ or (**F**) 0.5-mM NO_3_^–^ conditions. Error bars: SE (*n* = 10). Significant differences between transgenic lines and WT are indicated by asterisks (*p* < 0.05, one-way ANOVA).

**Figure S3.** The expressions of *OsPINs* in the roots of WT and *OsNRT2.1* transgenic lines under 0.5-mM NO_3_^−^ conditions with NPA treatment. WT and transgenic plants were shown in Figure 7. RNA was extracted from roots. Error bars: SE (*n* = 5). Significant differences between transgenic lines and WT are indicated by different letters (*p* < 0.05, one-way ANOVA).

**Figure S4.** ^15^NO_3_^−^ influx rate in roots of WT and *OsNRT2.1* transgenic lines under 0.5-mM ^15^NO_3_^−^ conditions. WT and transgenic plants. The seedlings were transferred to a quarter concentration of nutrient solution containing 0.5 mM of ^15^NO_3_^–^ for 5 min. Error bars: SE (*n* = 5). Significant differences between transgenic lines and WT are indicated by different letters (*p* < 0.05, one-way ANOVA).

**Figure S5.** Effects of NPA on root growth under 0.5-mM NH_4_^+^ or 0.5 mM NO_3_^−^ condition. Three-day-old normal grown *OE3/DR5::GUS* transgenic seedlings were transferred to nutrient solutions containing 1 μM of NPA from 100 mM of NPA that was dissolved in DMSO and the control containing the same amount of DMSO. Sampling was performed after seven days of treatment. (**A**) Scanning root morphology of rice plants under 0.5-mM NH_4_^+^ or 0.5 mM NO_3_^−^ conditions with or without NPA treatment. (bar = 1 cm); (**B**) GUS expression in the root tips of *OE3/DR5::GUS* seedlings under 0.5-mM NH_4_^+^ or 0.5-mM NO_3_^−^ conditions with or without NPA treatment. Root tips were stained for 2 h at 37 °C in the dark.
